# Supplementary material for: Using pressure to unravel the structure–dynamic-disorder relationship in metal halide perovskites
Source: Sci Rep. 2023 Jun 8;13:9300. doi: 10.1038/s41598-023-36501-w (PMC10250390; doi:10.1038/s41598-023-36501-w)
Supplement: Supplementary file 1 — Supplementary Information. [file 41598_2023_36501_MOESM1_ESM.pdf]

Supporting Information

Using Pressure to Unravel the Structure-Dynamic-Disorder Relationship in Metal Halide Perovskites

Kai Xu,[†] Luis Pérez-Fidalgo,[†] Bethan L. Charles,[‡,¶] Mark T. Weller,[‡,§] M. Isabel Alonso,[†] and Alejandro R. Goñi[*,†,||]

†*Institut de Ciència de Materials de Barcelona, ICMAB-CSIC, Campus UAB, 08193 Bellaterra, Spain*

‡*Dept. of Chemistry & Centre for Sustainable Chemical Technologies, University of Bath, Claverton Down, Bath BA2 7AY, UK*

¶*Dept. of Mechanical Engineering, Queens Building, University of Bristol, Bristol BS8 1TR, UK*

§*Dept. of Chemistry, Cardiff University, Wales CF10 3AT, UK*

||*ICREA, Passeig Lluís Companys 23, 08010 Barcelona, Spain*

E-mail: goni@icmab.es

$MAPbBr_3$ PL spectra & lineshape fitting

The main emission peak observed in the PL spectra of $MAPbBr_3$ at the temperatures and pressures of the experiments is assigned to free-exciton (FE) recombination.[1–4] Thus, for the quantitative analysis of the PL spectra we performed a lineshape fitting[5] using a Gaussian-Lorentzian cross-product function (lineshape fitting examples were published elsewhere[6]). Its expression reads as:

$$G \times L(\hbar\omega) = A \cdot \frac{\Gamma^2}{4 \cdot s \cdot (E_0 - \hbar\omega)^2 + \Gamma^2} \cdot \exp\left(-4 \cdot \ln 2 \frac{(1-s) \cdot (E_0 - \hbar\omega)^2}{\Gamma^2}\right) \tag{1}$$

where A is the amplitude prefactor, E_0 is the peak energy position, Γ is the full width at half maximum (FWHM) and s is a weight parameter which takes the value $s = 0$ for pure Gaussian lineshape and $s = 1$ for pure Lorentzian. The values of these four adjustable parameters were obtained as a function of temperature and/or pressure from lineshape fits to the measured PL spectra using Eq. (1). For the cases where there is a coexistence of two phases we used two cross-product functions with independent variable parameter sets. We note that the FE peak exhibits in $MAPbBr_3$ an asymmetry towards lower energies, being the left (low energy) half width at half maximum ca. 30% larger than the right (high energy) one. This has been taken into account using a split cross-product function like in Eq. (1) but with the corresponding half widths for photon energies below and above the peak position energy. The additional peaks apparent at low temperatures (below ca. 110 K) at lower energies than the FE peak are ascribed to radiative recombination of bound (acceptor/donor) exciton complexes, as published elsewhere.[7]

Figure S1 displays both the values of the FWHM (Γ) and the intensity of the main PL peak, extracted from the lineshape fits using the cross-product function of Eq. (1), plotted as a function of temperature for a $MAPbBr_3$ single crystal. With decreasing temperature down to 80 K the PL intensity increases exponentially almost at the same pace for the three crystal phases. This is the

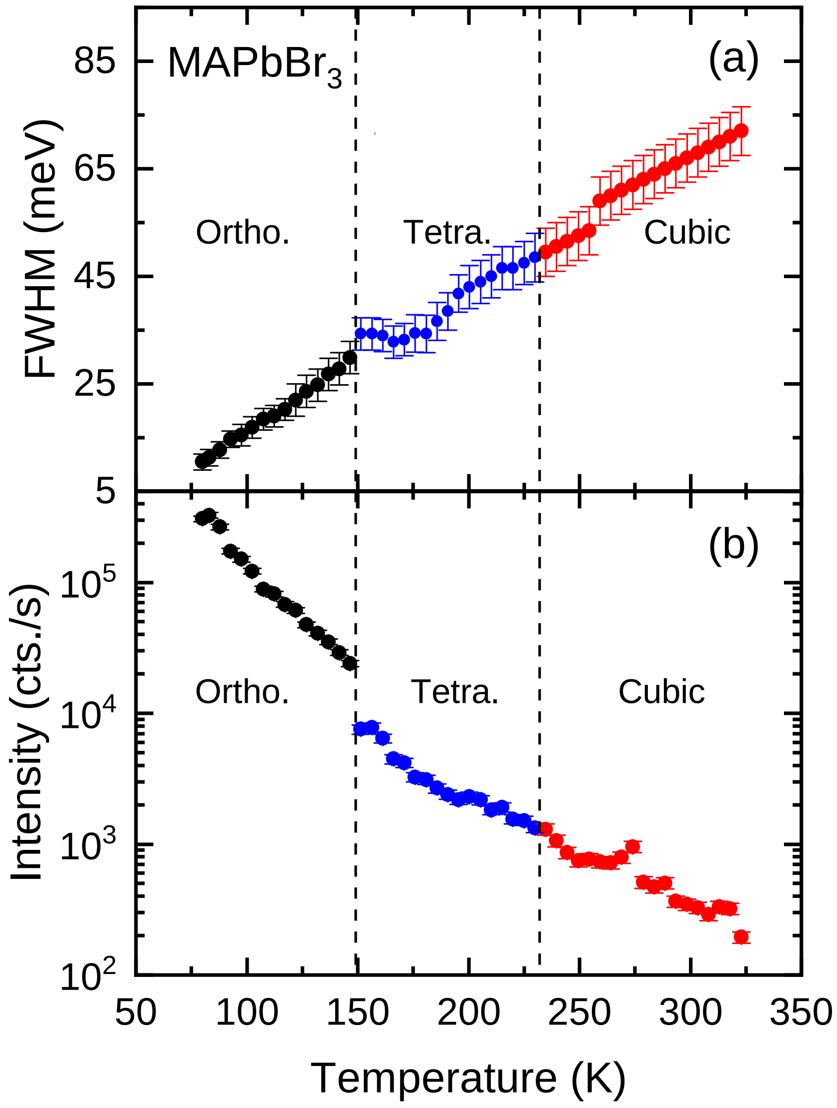

Figure S 1: (a) The PL FWHM (Γ) and (b) the peak intensity (*A*) plotted as a function of temperature, obtained from the PL lineshape fits using the cross-product function of Eq. (1) for a $MAPbBr_3$ single crystal. Different colors represent the different phases stable as a function of temperature, as indicated.

typical behavior of free-exciton radiative recombination as compared to bimolecular recombination of uncorrelated electron-hole pairs and is the result of an enhanced optical matrix element due to a reinforced Coulomb attraction, i.e. an increased wavefunction overlap between electron and hole forming the exciton. On the contrary, the exciton linewidth decreases (almost linearly) with decreasing temperature in the whole temperature range from 310 to 80 K. Such marked temperature dependence of the exciton linewidth is characteristic of an exciton broadening mechanism mediated by electron-phonon interaction given by:[9]

$$\Gamma(T) = \Gamma_0 + \frac{\gamma_{opt}}{e^{\frac{E_{opt}}{k_B \cdot T}} - 1}, \tag{2}$$

where Γ_0 is the temperature-independent inhomogeneous broadening which is mainly determined by the crystal quality, γ_{opt} is the electron-optical-phonon coupling constant and E_{opt} is an effective optical-phonon energy. Notice that the second term in Eq. (2) is nothing else than the Bose-Einstein occupation factor for a representative optical mode with an effective frequency, multiplied by an effective coupling strength constant. In Eq. (2) we have neglected the contribution stemming from exciton scattering by acoustic phonons, because the electron-acoustic-phonon coupling is often several orders of magnitude weaker than for optical phonons. In fact, Eq. (2) predicts a fairly linear temperature dependence of the exciton linewidth for temperatures higher that the effective temperature given by the effective optical-phonon energy E_{opt}. The latter is for MHPs typically in the range between 5 to 10 meV,[10–13] that means an effective temperature between 50 and 110 K. By performing the experiments down to liquid-nitrogen temperature, we miss completely the exponentially varying region described by Eq. (1). This would make a least-squares fit using Eq. (1) totally unreliable and for this reason we have not performed it for the data shown in Fig. S1a.

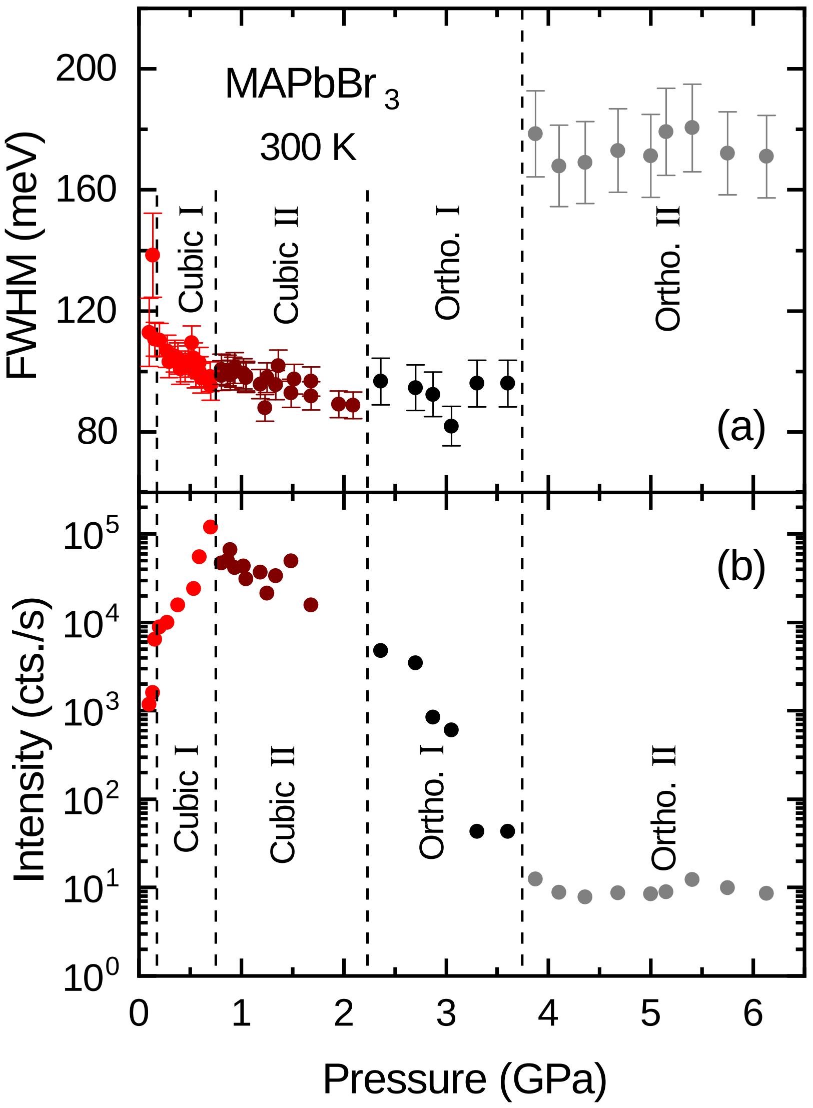

Figure S 2: (a) The PL FWHM (Γ) and (b) the peak intensity (*A*) plotted as a function of pressure, obtained from the PL lineshape fits using a cross-product function of Eq. (1) for a $MAPbBr_3$ single crystal. Different colors represent the different phases stable as a function of pressure, as indicated. The dashed vertical lines mark the different phase transition pressures.

Figure S2 shows the values of the FWHM (Γ) and the intensity of the main PL peak, extracted from the lineshape fits using the cross-product function of Eq. (1), plotted as a function of pressure for a $MAPbBr_3$ single crystal. The exciton linewidth decreases slightly with increasing pressure for

the three first phases, namely cubic-I, cubic-II and ortho-I. In contrast, the linewidth almost doubles in the high-pressure ortho-II phase, which is indicative of a pressure-induced inhomogeneity in the sample, probably due to an incipient amorphization. At the very beginning of the first stroke and in coincidence with the sudden redshift of the PL peak energy, both the linewidth decreases and the intensity increases abruptly. We speculate that this behavior results from strain relaxation effects the first time the small chip of the sample is pressurized in the DAC. The overall intensity of the PL emission decreases with increasing pressure by several orders of magnitude, except for the cubic-I phase, for which the PL intensity increases strongly with pressure.

$MAPbBr_3$ Raman spectra

Figure S3 display a series of raw Raman spectra measured in the low-frequency spectral range of the inorganic cage modes at different pressures and at room temperature. The different colors indicate the different high-pressure phases adopted by $MAPbBr_3$ under compression. These are the raw data, except for a normalization to the maximum intensity to ease their comparison. No background function was subtracted. The gradual decrease in linewidth of the Raman peaks for the subsequent high pressure phases can be clearly appreciated in the spectra of Fig. S3.

Homogeneous vs. inhomogeneous broadening

Assuming that the resolution of the Raman spectrometer is sufficiently high, such that there is no instrumental broadening, the linewidths of the Raman peaks are basically determined by two contributions: The *homogeneous* broadening, given by the inverse of the phonon lifetime, and the *inhomogeneous* broadening, which arises a from inhomogeneity in the sample leading to a local distribution of bond lengths. At the origin of the homogeneous linewidth is just the uncer-

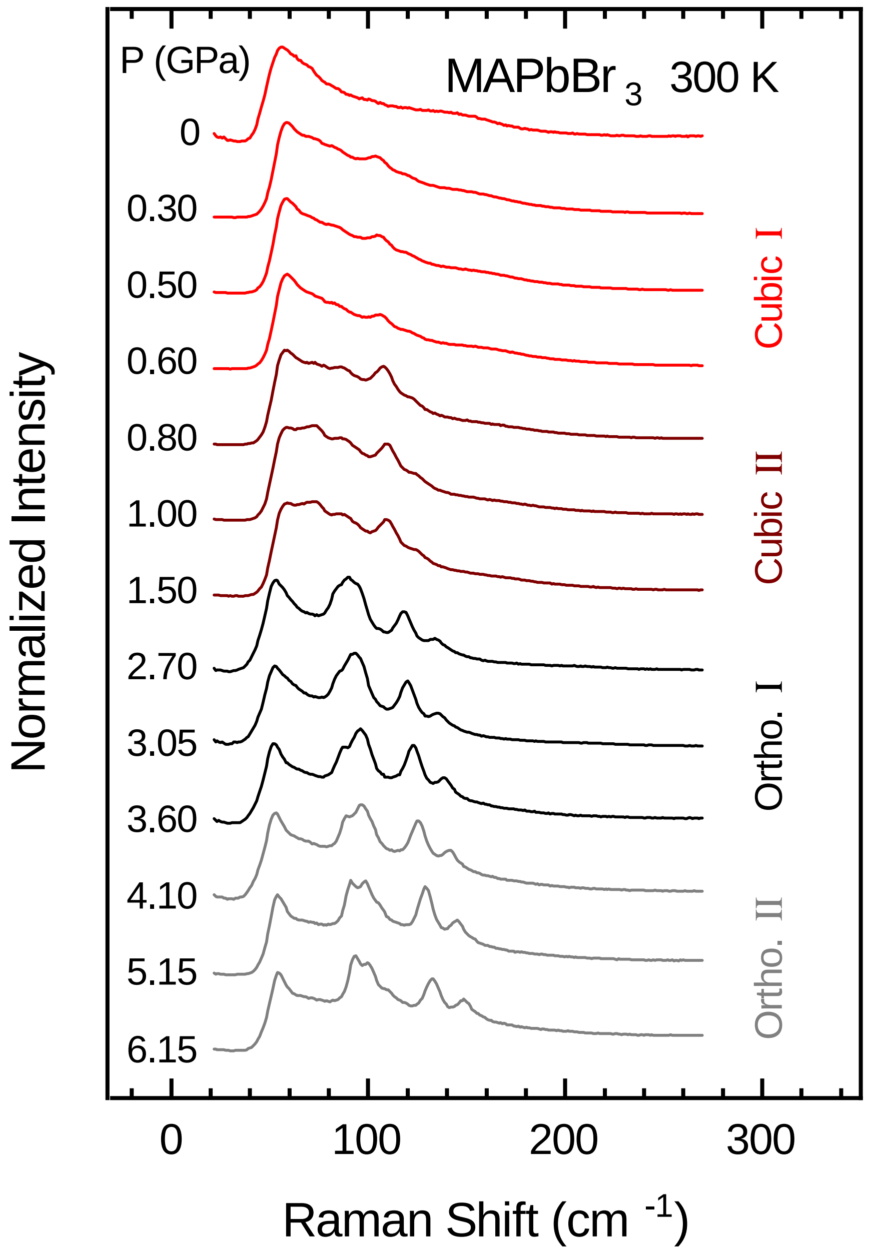

Figure S 3: Raw Raman spectra of $MAPbBr_3$ recorded at different pressures and at room temperature in the spectral range of the inorganic cage phonons using the 785-nm line for excitation. The spectra were normalized to their maximum intensity and shifted vertically for clarity. The different colors of the spectra correspond to the different phases adopted by $MAPbBr_3$ under compression.

tainty principle which states that the uncertainty in energy (phonon linewidth) increases in inverse proportion to the uncertainty in time (phonon lifetime), that is $\Gamma \propto \frac{1}{\tau}$. Time-dependent quantum mechanics tells us that the lineshape of homogeneously broadened Raman peaks is a Lorentzian function, as illustrated by the sketch in Fig. S4. The very fact of a finite lifetime implies that there is a certain perturbation that causes a sizable anharmonicity in the vibrational (phononic) spectrum of the material considered. In good quality crystals, anharmonicities are mainly due to phonon-phonon scattering. Since the strength of the phonon-phonon interaction increases with the phonon population, the homogeneous linewidth Γ is strongly temperature dependent. The peculiarity of metal halide perovskites (MHPs) is that the leading phonon-phonon interaction arises from the coupling between the inorganic cage and the A-site cations, as we show further below, rather than from "internal" scattering processes among the phonon modes of the inorganic cage alone.

In contrast, inhomogeneous linewidths have a totally different origin. Phonons are collective excitations (vibrations) of the crystal lattice whose frequencies are largely determined by the characteristics of the bonds in the crystal structure. Any inhomogeneity in a single crystal that would locally generate strain or lattice deformation, would also induce inhomogeneous broadening of the phonons. In the inhomogeneous crystal, the distribution of bond lengths, i.e. of bond strengths, created by the inhomogeneity leads also to a distribution of phonon frequencies $\Delta\omega$ (see Fig. S4). A "normal" distribution of frequencies produces a Gaussian lineshape in the Raman spectrum with a FWHM given by the inhomogeneous broadening, provided that $\Delta\omega > \Gamma$. The corresponding sketch in Fig. S4 illustrates the process by which the inhomogeneous linewidth develops: The Gaussian lineshape corresponds to the convolution of the Lorentzian peaks representing the normal distribution of frequencies. Since such distribution does not change with temperature, inhomogeneous linewidths are fairly temperature insensitive.

We now turn to the discussion of a striking systematic that concerns the linewidths of the

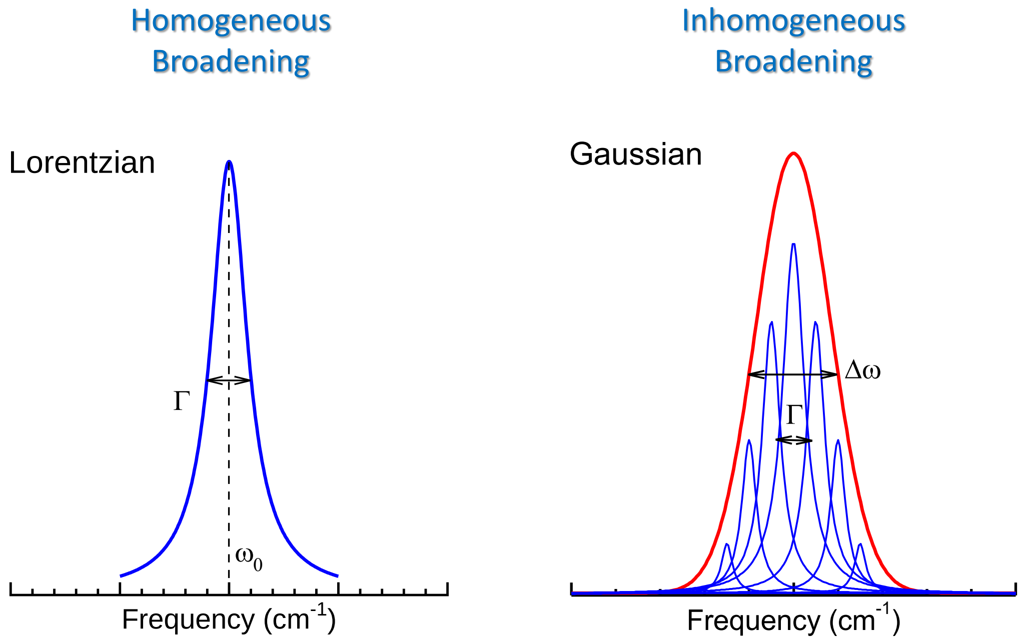

Figure S 4: Schematic representation of the lineshape profile of an homogeneously (Lorentzian) and inhomogeneously (Gaussian) broadened Raman peak.

Raman peaks associated with the inorganic cage phonons as a function of temperature, observed for the three methylammonium lead halide perovskites ($MAPbX_3$ with X= Cl, Br, and I). The supporting experimental data correspond to the Raman spectra shown in Figs. 8a, 8f, and 8k of the work of Leguy *et al.*[8] In summary, from the Raman spectra recorded in the spectral region below ca. 200 cm^{-1} of the inorganic cage phonons, one can make following observations: i) For $MAPbI_3$ the Raman spectra of the cubic and tetragonal phases, where the MA dynamics is unfolded, are broad and almost featureless, whereas in the low-temperature orthorhombic phase the Raman peaks are well-defined and much sharper. ii) The other extreme is $MAPbCl_3$ for which the Raman spectra exhibit fairly broad peaks at all temperatures, i.e. for all three phases, although there is a slight but clear temperature dependence of the linewidth. iii) $MAPbBr_3$ is an intermediate case between the

other two, being the difference in linewidth of the Raman peaks between the orthorhombic phase and the other two phases much less marked than for $MAPbI_3$. These results are summarized in the table of Fig. 5, where the red arrows represent the magnitude of the line broadening in each case.

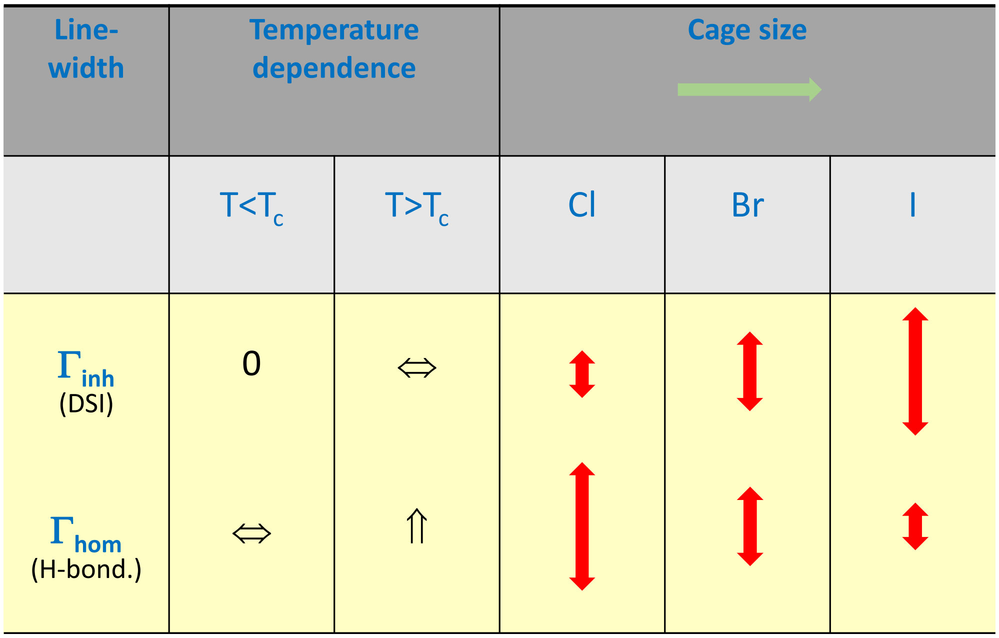

Figure S 5: Table summarizing the relative magnitude (red arrows length) of the homogeneous and inhomogeneous broadening contributions to the linewidth for different inorganic cage sizes, below or above the temperature (T_C) at which the MA molecules become locked inside the cage voids.

As we argue in the main manuscript, for $MAPbI_3$ the dynamic steric interaction (DSI) is the cause of the large inhomogeneous broadening of the inorganic cage phonons in the cubic and tetragonal phases, because of the dynamic disorder induced by the unleashed MA dynamics. In the orthorhombic phase, the MA molecules become locked within the cage voids in a state of static order, hence the inhomogeneous broadening vanishes and the linewidths are then determined by the homogeneous part. For $MAPbI_3$ it turns out that the inhomogeneous broadening overwhelms the homogeneous contribution ($\Gamma_{inh.} \gg \Gamma_{hom.}$). Since with locked A-site cations there is no DSI, the coupling between both sublattices is mediated by H-bonding interaction. The striking result

is that for MHPs, the homogeneous linewidth, that is, the phonon lifetime, is determined by the H-bonding interaction and not by anharmonicities of the inorganic cage itself. The signature of H-bonding is seen in the clear dependence on halide atom of the homogeneous linewidth of the peaks in the Raman spectra of the orthorhombic phases. As shown in the table of Fig. 5 and in Figs. 8a, 8f, and 8k of Leguy *et al.*,[8] the phonon linewidth increases with decreasing cage void volume for the compound sequence $MAPbI_3$→$MAPbBr_3$→$MAPbCl_3$. For the $MAPbCl_3$ the cage voids are so tight that the H-bonding mediated coupling between the inorganic cage phonons and the MA vibrations leads to a loss of coherence of the former, which in turn impacts the phonon lifetime. The result is that for $MAPbCl_3$ the homogeneous broadening surpasses in magnitude the inhomogeneous contribution ($\Gamma_{inh.} \ll \Gamma_{hom.}$). For this reason the Raman spectra of $MAPbCl_3$ are broad at all temperatures, irrespective if the MA molecules are locked or free to move. Again, $MAPbBr_3$ is an intermediate case, for which holds that $\Gamma_{inh.} \geq \Gamma_{hom.}$. This is why in the high pressure experiments the reduction of the phonon linewidths, when $MAPbBr_3$ transforms into the here reported high-pressure orthorhombic phases, is not so pronounced as for $MAPbI_3$.[6]

References

(1) D'Innocenzo, V.; Grancini, G.; Alcocer, M. J.-P.; Kandada, A. R. S.; Stranks, S. D.; Lee, M. M.; Lanzani, G.; Snaith, H. J.; Petrozza, A. Excitons Versus Free Charges in Organo-Lead Tri-Halide Perovskites. *Nature Commun.* **2014**, 5, 3486-3492.

(2) Wu, K.; Bera, A.; Ma, C.; Du, Y.; Yang, Y.; Li, L.; Wu, T. Temperature-Dependent Excitonic Photoluminescence of Hybrid Organometal Halide Perovskite Films. *Phys. Chem. Chem. Phys.* **2014**, 16, 22476-22481.

(3) Dong, Q.; Fang, Y.; Shao, Y.; Mulligan, P.; Qiu, J.; Cao, L.; Huang, J. Electron-Hole Dif-

fusion Lengths >175 μm in Solution-Grown $CH_3NH_3PbI_3$ Single Crystals. *Sci.* **2015**, 347, 967-970.

(4) Galkowski, K.; Mitioglu, A. A.; Surrente, A.; Yang, Z.; Maude, D. K.; Kossaki, P.; Eperon, G. E.; Wang, J. T.-W.; Snaith, H. J.; Plochocka, P.; et al. Spatially Resolved Studies of the Phases and Morphology of Methylammonium and Formamidinium Lead Tri-Halide Perovskites. *Nanoscale* **2017**, 9, 3222-3230.

(5) Wojdyr, M. Fityk: A General-Purpose Peak Fitting Program. *J. Appl. Crystallogr.* **2010**, 43, 1126-1128.

(6) Francisco-López, A.; Charles, B.; Weber, O. J.; Alonso, M. I.; Garriga, M.; Campoy-Quiles, M.; Weller, M. T.; Goñi, A. R. Pressure-Induced Locking of Methylammonium Cations Versus Amorphization in Hybrid Lead Iodide Perovskites. *J. Phys. Chem. C* **2018**, 122, 22073-22082.

(7) Francisco-López, A.; Charles, B.; Alonso, M. I.; Garriga, M.; Weller, M. T.; Goñi, A. R. Photoluminescence of Bound-Exciton Complexes and Assignment to Shallow Defects in Methylammonium/Formamidinium Lead Iodide Mixed Crystals. it Adv. Optical Mater. **2021**, 2001969/1-9.

(8) Leguy, A. M. A.; Goñi, A. R.; Frost, J. M.; Skelton, J.; Brivio, F.; Rodríguez-Martínez, X.; Weber, O. J.; Pallipurath, A.; Alonso, M. I.; Campoy-Quiles, M.; Weller, M. T.; Nelson, J.; Walsh, A.; Barnes, P. R. F. Dynamic Disorder, Phonon Lifetimes, and the Assignment of Modes to the Vibrational Spectra of Methylammonium Lead Halide Perovskites. *Phys. Chem. Chem. Phys.* **2016**, 18, 27051-27066.

(9) Rudin, S.; Reinecke, T. L.; Segall, B. Temperature-Dependent Exciton Linewidths in Semiconductors. *Phys. Rev. B* **1990**, 42, 11218-11231.

(10) Diab, H.; Trippé-Allard, G.; Lédée, F.; Jemli, K.; Vilar, Ch.; Bouchez, G.; Jacques, V. L. R.; Tejeda, A.; Even, J.; Lauret, J.-S.; *et al.* Narrow Linewidth Excitonic Emission in Organic-Inorganic Lead Iodide Perovskite Single Crystals. *J. Phys. Chem. Lett.* **2016**, 7, 5093-5100.

(11) Wright, A. D.; Verdi, C.; Milot, R. L.; Eperon, G. E.; Pérez-Osorio, M. A.; Snaith, H. J.; Giustino, F.; Johnston, M. B.; Herz, L. M. Electron-Phonon Coupling in Hybrid Lead Halide Perovskites. *Nature Commun.* **2016**, 7, 11755/1-9.

(12) Phuong, L. Q.; Nakaike, Y.; Wakamiya, A.; Kanemitsu, Y. Free Excitons and Exciton-Phonon Coupling in $CH_3NH_3PbI_3$ Single Crystals Revealed by Photocurrent and Photoluminescence Measurements at Low Temperatures. *J. Phys. Chem. Lett.* **2016**, 7, 4905-4910.

(13) A. Francisco-López, B. Charles, M. I. Alonso, M. Garriga, M. Campoy-Quiles, M. T. Weller, A. R. Goñi, Phase Diagram of Methylammonium/Formamidinium Lead Iodide Perovskite Solid Solutions from Temperature-Dependent Photoluminescence and Raman Spectroscopies. *J. Phys. Chem. C* **2020**, 124, 3448-3458.
